# Estimation of global tropical cyclone wind speed probabilities using the STORM dataset

**DOI:** 10.1038/s41597-020-00720-x

**Published:** 2020-11-10

**Authors:** Nadia Bloemendaal, Hans de Moel, Sanne Muis, Ivan D. Haigh, Jeroen C. J. H. Aerts

**Affiliations:** 1 Institute for Environmental Studies (IVM), Vrije Universiteit Amsterdam, 1081 HV Amsterdam, the Netherlands; 2 Deltares, 2600 MH Delft, The Netherlands; 3 School of Ocean and Earth Science, National Oceanography Centre, University of Southampton, European Way, Southampton, SO14 3ZH United Kingdom

**Keywords:** Natural hazards, Atmospheric dynamics

## Abstract

Tropical cyclones (TC) are one of the deadliest and costliest natural disasters. To mitigate the impact of such disasters, it is essential to know extreme exceedance probabilities, also known as return periods, of TC hazards. In this paper, we demonstrate the use of the STORM dataset, containing synthetic TCs equivalent of 10,000 years under present-day climate conditions, for the calculation of TC wind speed return periods. The temporal length of the STORM dataset allows us to empirically calculate return periods up to 10,000 years without fitting an extreme value distribution. We show that fitting a distribution typically results in higher wind speeds compared to their empirically derived counterparts, especially for return periods exceeding 100-yr. By applying a parametric wind model to the TC tracks, we derive return periods at 10 km resolution in TC-prone regions. The return periods are validated against observations and previous studies, and show a good agreement. The accompanying global-scale wind speed return period dataset is publicly available and can be used for high-resolution TC risk assessments.

## Introduction

Tropical cyclones (TCs) are amongst the deadliest and costliest natural disasters, affecting people, economies and the environment in coastal areas around the globe. In 2019, Cyclone Idai caused over 1,000 fatalities and displaced 3 million people upon landfall in Mozambique^1^. In 2017, Hurricanes Harvey, Irma and Maria entered the top-5 costliest Atlantic hurricanes ever, with combined losses estimated at $220 billion^2^. To minimize future loss of life and property, it is crucial to perform accurate TC risk assessments and identify high-risk locations so that appropriate protection measures can be designed.

Wind is one of the major hazards associated with TCs and can do substantial damage to housing, infrastructure and ecosystems both in coastal regions and far inland^3^. Moreover, wind correlates with the intensity of other TC-induced hazards, such as storm surges, waves and precipitation^4–6^. To enhance our understanding of TC risk at the global scale, it is therefore essential to analyze wind speed probabilities in coastal zones. Risk is commonly calculated as the integrated value of expected damages over all exceedance probabilities –the inverse of these probabilities being return periods (RPs)^7^. As such, accurately calculating risk requires information on a wide range of RPs. Simpson and Lawrence^8^ empirically estimated TC RPs along 80 km-long coastal segments of the US coastline based on historical TCs. However, RPs could not be calculated for those coastal segments that were not hit by a TC in the 85 years of observations. This shows that, due to the short length of the observational record, data often needs to be aggregated over larger spatial regions to perform a RP analysis, hereby omitting the spatial heterogeneity. Moreover, estimating RPs comes with large uncertainties, especially for RPs exceeding the length of the observational record. To overcome these limitations, the methodology of synthetic TC track generation has been developed over the past few decades^9–12^. In this approach, TCs, extracted from either historical data^9,13^ or climate model simulations^14^, are statistically resampled and modeled to generate synthetic, but realistic, TCs. Using a Monte Carlo approach, this procedure is repeated recurrently to construct a TC dataset having the same statistical characteristics as the input dataset, but spanning hundreds to thousands of years.

Using synthetic data enables the analysis of higher RPs and at higher spatial resolution than previously possible. In an accompanying paper we have presented the global synthetic model STORM (Synthetic Tropical cyclOne geneRation Model)^12^. The STORM dataset spans 10,000 years of global TC activity under present-day climate conditions, based on observed TC tracks. Here, we demonstrate usage of the STORM dataset by creating wind speed RPs at three different spatial scales: (i) basin level; (ii) within 100 km for 18 selected coastal cities and 63 islands; (iii) at 10 km resolution in TC-prone regions. This dataset is unique in presenting (high) RPs at a global scale for all TC-prone regions. More importantly, it represents an important step forward to calculating global TC damages and risk.

## Results

### Deriving return periods from the STORM dataset

Calculating RPs of (extreme) wind speeds in the STORM dataset can be done either empirically or statistically. When using an empirical approach, RPs are directly calculated from wind speeds ranked in order of magnitude using formulas like Hazen’s or Weibull’s plotting formula^15,16^. A benefit of this approach is that no specific shape of the RP-curve is assumed; RPs are calculated per given wind speed without interpolation or smoothening of the data. However, the highest RP is limited by the temporal length of the data as this method does not allow for extrapolation beyond this timespan. TC risk assessments typically require information on extreme events that have not been observed yet. Therefore RPs are often determined by fitting extreme value (EV) distributions^17^ to historical data. This way any RP can be estimated, even those beyond the range of observations. To ensure there is enough data for a good fit, this approach is generally carried out for ocean basins or relatively large coastal sections. Such fitted RPs are strongly influenced by the selected EV distribution, especially for higher RPs^18^. Furthermore, short records have large uncertainties, and typically multi-decadal records are needed for reliable estimates of the tail (high RPs)^19^.

Here we compare these two approaches to estimate basin-scale RPs using the synthetic TCs from STORM. We apply Weibull’s plotting formula to the maximum 10-meter 10-minute average sustained wind speeds (max U10) in the full STORM dataset (10,000 years). We use max U10 because this is globally the most commonly reported value of wind speeds^20^, but other averaging periods can easily be obtained using conversion factors^21^. Next, we fit five EV distributions (the generalized extreme value, exponential, Gumbel, Weibull and Pareto distribution) to 1,000 random realizations of 38 years of data sampled from the STORM dataset. This 38-year length was chosen as the STORM dataset was created using 38 years of historical data (1980–2017) from the International Best Track dataset for Climate Stewardship (IBTrACS^20^).

**Fig. 1:**
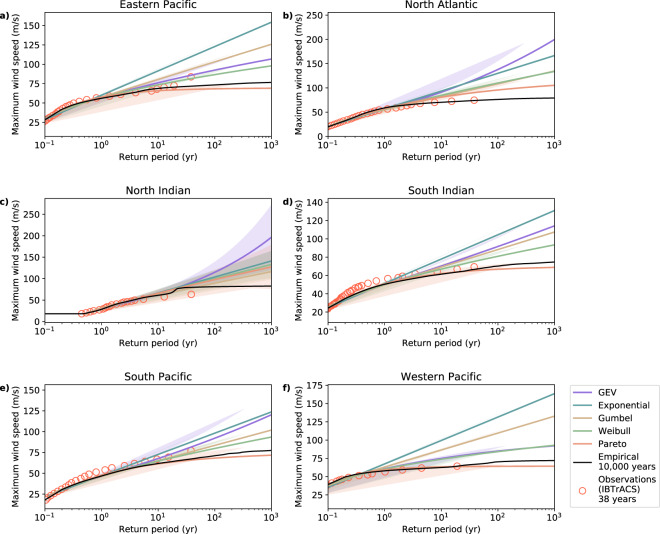
Comparison of the wind speed return periods based on fitting five different extreme value distributions to 1,000 random realizations of 38 years and applying an empirical distribution (the Weibull plotting formula) to the full 10,000 years of data. Data is aggregated at basin level for each of the 6 ocean basins (**a**–**f**). The extreme value distributions are the generalized extreme value distribution (purple), the exponential distribution (blue), the Gumbel distribution (also known as the Generalized Extreme Value Distribution Type-I; yellow), the Weibull distribution (also known as the Generalized Extreme Value Distribution Type-II; green), and the Pareto distribution (red). Shaded areas indicate the 95%-confidence interval based on the bootstrap with 1,000 realizations. Empirically derived return periods from observations (IBTrACS) are given as red scatter points. See Methods for a full description of the basin domains.

At basin-level, the empirically derived STORM-RPs agree with the observed RPs (Fig. 1). In the North Atlantic, North Indian, and Western Pacific, STORM-RPs compare well with observations. In the Eastern Pacific and the Southern Hemisphere basins, max U10 in STORM are lower than the highest observed counterparts. Additionally, for four out of five EV distributions the max U10 values are substantially higher than the empirically derived values, particularly at RPs exceeding 100-yr (Fig. 1). Compared to the empirical curve, the worst-performing EV distributions are the exponential, generalized extreme value (GEV) and Gumbel distributions, which deviate from the empirical curve above the 10-yr RP. The EV distributions cannot capture the asymptotic behavior of the TC intensity, caused by environmental constraints such as the Maximum Potential Intensity^22,23^. Consequently, at high RPs, max U10 from EV distributions are higher than their empirically derived counterparts, with a maximum difference of 117 m/s for the GEV in the North Atlantic at the 1,000-yr RP. For the North Indian, we observe a kink in the empirical curve around the 20-yr RP: this is likely caused by an absence of certain max U10 in the dataset, which are then excluded from the RP calculation. This absence is driven by a limited spatial distribution of sea-surface temperatures (SSTs), causing a more frequent occurrence of higher max U10. Figure 1 shows that the Pareto distribution is the best-performing EV distribution compared to the empirical approach. However, for the North Indian, also the Pareto distribution shows substantially higher RPs compared to the empirical curve. Overall, the empirical probabilities of the observations are well in line with the estimates from the STORM dataset, and are considerably lower than using EV fits at RPs exceeding 100-yr.

### Tropical cyclone return periods for coastal cities

**Fig. 2:**
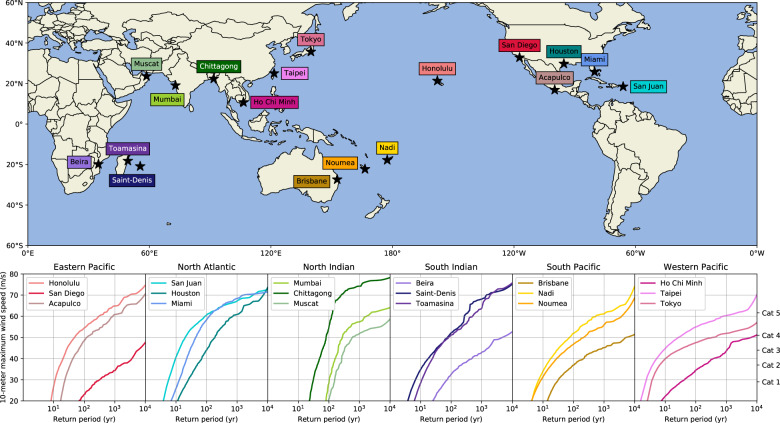
Return periods of maximum wind speed (10-minute 10-meter average) within a radius of 100 km for a selection of coastal cities. Color tones correspond to the different basins: North Atlantic (blue), Eastern Pacific (red), Western Pacific (pink), North Indian (green), South Indian (purple), and South Pacific (yellow). Graphs show the return period on the x-axis versus the corresponding maximum wind speed (in m/s) on the y-axis. Category-classifications are based on the Saffir-Simpson scale (converted from 1-min to 10-min thresholds, see Methods).

Besides basin-scale RPs, we derive RPs for specific coastal locations using a 100 km radius to capture those TCs that have a substantial impact. We demonstrate this here for 18 coastal cities, but a similar dataset is available for 63 islands (see Data availability Statement). The RP-curves (subplots in Fig. 2; see Table 1) show that probabilities of a TC event occurring within 100 km differ strongly per city. Cities that are not regularly hit by a TC include San Diego (USA), Mumbai (India), and Muscat (Oman) with RPs for a Category-1 exceeding 100-yr. These relatively high RPs are driven by multiple TC characteristics. Firstly, TCs are generally deflected from San Diego and Mumbai^24^ and instead move out over the open ocean. This is caused by TCs being embedded within the prevailing easterly (westward) flow at these latitudes. Secondly, TCs dissipate when they make landfall. As Muscat is located in the relatively narrow Gulf of Oman, most TCs in this region will likely have passed land upon approaching Muscat. Lastly, both Mumbai and Muscat lie in the North Indian, where approximately 2 TCs form per year^12^, hereby further decreasing the chances of being hit by a TC in any given year. Of the cities considered here, Taipei (Taiwan) and Tokyo (Japan) experience TCs most often, with RPs for a Category-1 lower than 4-yr. Both cities are located in the Western Pacific, the most active basin with 22.5 TC formations per year^12^. Of the cities considered, San Juan (Puerto Rico) and Chittagong (Bangladesh) are most often affected by strong TCs, with Category-5 TCs having a 131-yr RP for both cities. San Juan’s central position in the tropical Atlantic Ocean combined with frequent Category-5 TC formations (approximately 1-in-6-years; see Fig. 1) likely drives these relatively low RPs. Chittagong’s relatively low RP is likely due to the high SSTs in the Bay of Bengal, enhancing TC intensification and thus generating strong TCs.

**Table 1:** Return periods (yr) of Category 1–5 wind speeds occurring within 100 km for 18 coastal cities.

City	Country	Cat 1 (29 m/s)	Cat 2 (37.6 m/s)	Cat 3 (43.4 m/s)	Cat 4 (51.1 m/s)	Cat 5 (61.6 m/s)
Honolulu	Hawaii (USA)	11	17	25	54	442
San Diego	USA	247	1870	5034	>10,000	>10,000
Acapulco	Mexico	22	35	62	156	1684
San Juan	Puerto Rico	5	8	12	22	131
Houston	USA	23	52	92	205	1230
Miami	USA	12	20	28	48	155
Mumbai	India	101	140	199	285	2403
Chittagong	Bangladesh	30	44	59	83	131
Muscat	Oman	151	235	315	768	>10,000
Beira	Mozambique	64	238	1179	6817	>10,000
Saint-Denis	Réunion	6	13	28	87	347
Toamasina	Madagascar	10	19	31	101	616
Brisbane	Australia	28	111	466	9013	>10,000
Nadi	Fiji	7	14	27	87	1093
Noumea	New-Caledonia	8	20	51	263	4755
Ho Chi Minh	Vietnam	33	225	970	9673	>10,000
Taipei	Taiwan	2	4	8	36	2200
Tokyo	Japan	4	7	23	556	>10,000

Return periods are derived from the STORM dataset. Wind speeds are given as 10-meter 10-minute sustained average values, see Methods.

### Spatial distribution of extreme wind speeds

Using STORM, we can also derive RPs at high (10 km) spatial resolution. While a single extreme event might be captured well in historical datasets (e.g. Hurricane Irma in IBTrACS), there are not enough events in such datasets to robustly calculate high RPs. To calculate max U10 at 10 km resolution, we fit a 2D-parametric wind model to each synthetic TC (see Methods). Note that RPs inherently depend on the spatial scale they are computed at. At basin-scale, multiple TCs form every year, each one potentially reaching a given max U10. At a high-resolution grid cell, a TC passage can be rare. Hence, for equal max U10, RPs are lower when computed at higher resolution.

**Fig. 3:**
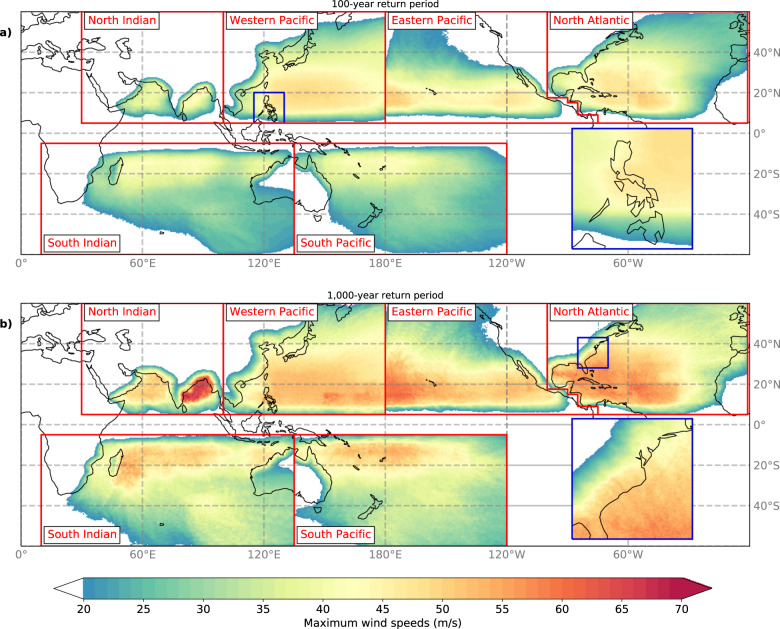
Spatial distribution of 10-meter 10-minute sustained maximum wind speeds (m/s) at 10 km resolution, derived from applying a 2D-wind parametrization to the synthetic tropical cyclone tracks in STORM. The wind speeds are the average value of 1,000 random realizations of 10,000 years of data (sampled with replacement) and determining RPs using Weibull’s plotting formula to each realization, performed at each coordinate at the 1-in-100-year (**a**) and the 1-in-1,000-year return period (b), respectively. The return period-analysis is carried out at the basin scale: as such, there is often no smooth transition of wind speed values at the basin boundaries. Inset figures show the distribution of wind speeds around the Philippines (**a**) and the United States East Coast (**b**) at the given return period.

Figure 3 shows that max U10 increases between the 100-yr and 1,000-yr RP level, with largest increases in the Bay of Bengal, the North Atlantic, and west of Hawaii in the Eastern Pacific, and less profound in the mid-latitudes and over land. There is distinct spatial variation within basins driven by the strong relationship between SSTs and TC activity. This is for instant evident in the Bay of Bengal, where SSTs^25^ of approximately 29 °C drive TC intensification, resulting locally in max U10 exceeding 65 m/s for a 1,000-yr RP. Aside from basin-scale variability in max U10, the two insets in Fig. 3 display the variability in max U10 at smaller scales. Distinct differences in max U10 are visible around the Philippines (inset Fig. 3a), with lower max U10 on the east side caused by the (westward) passage of TCs over the archipelago. Along the US coastline (inset Fig. 3b), stronger TCs make landfall more frequently on the Florida-North Carolina coastline than around New York City (NYC). This is driven by two factors: (i) TCs generally move north-westward near the Florida-North Carolina regions (onshore direction), whereas TCs are defected north-eastward near NYC (offshore direction); and (ii) SSTs are higher along the southern coastline, supporting intense TCs, whilst the lower SSTs around NYC drive a weakening of TCs. The apparent re-intensification of TCs near 40°S is partly caused by relatively high SSTs of 17–22 °C. Another cause is that these mid-latitude regions are mostly dominated by extratropical cyclones, which follow a different intensification process than TCs. STORM, however, does not model the extratropical transition of TCs and as such may underestimate RPs in these regions. Another feature visible in predominantly the mid-latitudes is the dotted patterns (e.g. North of Hawaii, Fig. 3). These patterns are caused by the passage of few TCs, combined with a higher translational speed at these latitudes. As we use 3-hourly intervals, the max U10 values appear as dots.

### Spatial distribution of return periods of tropical cyclones

**Fig. 4:**
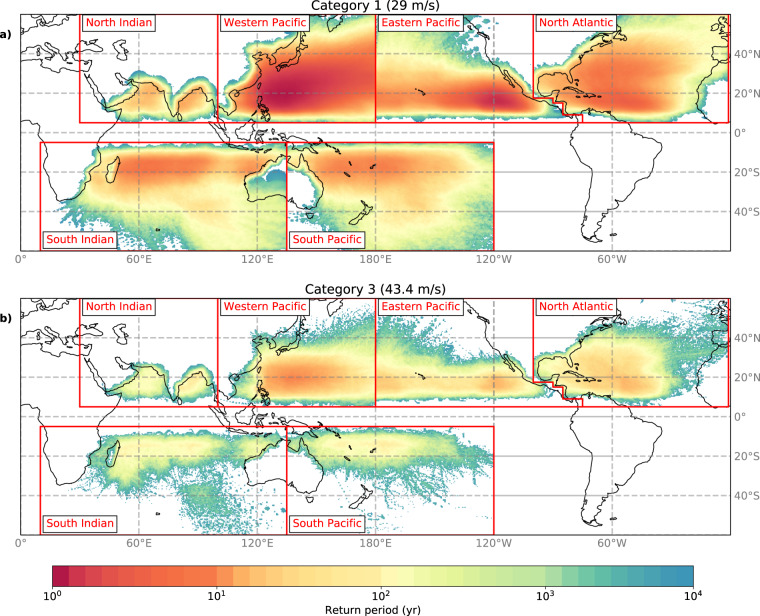
Spatial distribution of return periods (yr) at 10 km resolution, derived from applying a 2D-wind parametrization to the synthetic tropical cyclone tracks in STORM. The return periods are the average value of 1,000 random realizations of 10,000 years of data (sampled with replacement) and determining RPs using Weibull’s plotting formula to each realization at each coordinate at Category 1 (wind speeds ≥ 29 m/s) (**a**), and Category 3 tropical cyclone strength (wind speeds ≥ 43.4 m/s) (**b**), respectively.

Besides calculating max U10 for specific RPs, we can also reverse the procedure and compute the RP for given TC-categories. Figure 4 illustrates the RPs of a Category-1 (max U10 ≥ 29 m/s) and a Category-3 TC (max U10 ≥ 43.4 m/s) on the converted Saffir-Simpson Scale^26^, see Methods. There are large spatial variations, but for all basins RPs are lowest for a Category-1 or Category-3 event in the tropical regions. For large parts of the Western Pacific and the eastern part of the Eastern Pacific, Category-1 TCs have an approximate 2-yr RP. In the other basins, these RPs lie between 5 and 20-yr. Category-3 TCs, however, occur less frequent, ranging between 10-yr RP for the Western Pacific to 90-yr RP for the North Indian.

## Discussion

### Comparison to other synthetic datasets

At basin-level, we have shown that the STORM-RPs compare well with observations (Fig. 1). At local scale, however, data can be scarce. Additionally, the observational dataset used here only spans 38 years, making it unfit for RP analysis past this timespan. Hence, here we compare our results to other studies that derived RPs based on thousands of years of synthetic TC tracks. We first compare model outcomes at the local scale, after which we discuss global-scale patterns in RPs.

For 18 cases we compare max U10 for given RPs with STORM, and for Mumbai we compare the RPs for given categories (see Online-only Table 1). Sobel, *et al*.^24^ reported a 49 to 97-yr RP for a Category-1 TC within 150 km from Mumbai, agreeing well with the STORM-RP of 66-yr. Similarly, they estimate Category-3 RPs around 500-yr, while STORM-RPs are around 138-yr. For a Category-1 TC occurring in Mumbai, Sobel, *et al*.^24^ list a 224 to 236-yr RP; compared to a STORM-RP of 95-yr. For the city itself, RPs vary between 3,000 to 10,000-yr, compared to 550-yr in STORM. STORM-RPs are predominantly lower than those in Sobel, *et al*.^24^. Sobel, *et al*.^24^, however, speculate that they underestimate the TC hazard. Further differences are likely driven by the use of different track modeling methods, wind field parameterization schemes (including different resolutions) and RP estimation techniques.

STORM performs well for given RPs: for 13 out of 18 cases, absolute differences between max U10 in STORM and other models lie within 5 m/s. The largest difference is 18 m/s for Darwin, Australia^27^. This relatively large difference (−38.3%) is likely caused by the fact that Darwin is located near the South Indian-South Pacific basin border, and is hit by TCs originating in both basins. STORM, however, models TCs per basin, cutting off South Pacific TCs at the basin boundary. Townsville and Port Hedland are located further away from the basin boundary and max U10 are in better agreement. Additionally, we observe relative differences of −35.9% and −25.1% for the Federated States of Micronesia (FSM) and Palau, respectively. In STORM, the lower basin boundary in the Western Pacific is set at 5°N. As these island countries lie at around 7°N, and modelled TCs generally deflect away from the Equator, this means most TCs in STORM pass north of the island countries. Conversely, the AIR Tropical Cyclone Model^28^ models the Pacific region as a whole, and thus TCs have a higher probability of affecting FSM and Palau.

At the global scale, Lee, *et al*.^29^ calculated RPs for Category-1 and 3 TCs using the CHAZ model. The general spatial patterns in the CHAZ model (Figure 12–13 in Lee, *et al*.^29^) and STORM (Fig. 4) agree well, particularly in the Western and Eastern Pacific, including low RPs (around 1 to 10-yr for Category-1 and in the order of 10-yr for Category 3) in the Western and Eastern Pacific. For the North Atlantic and North Indian, STORM-RPs are lower than CHAZ-RPs. However, Lee, *et al*.^29^ illustrate that their RPs are higher in the North Atlantic than observations. In parts of the Southern basins, STORM-RPs are around 90-yr while CHAZ-RPs are approximately 10 to 50-yr for a Category-3.

In conclusion, the STORM-RPs show good agreement with other studies, with differences in max U10 for a given RP often being less than 5 m/s. At the global scale, we observe similar spatial patterns of RPs for a Category-1 TC, but deviations at smaller scales occur when assessing Category-3 RPs.

### Limitations and future research directions

In previous sections, we have demonstrated that the STORM-RPs for max U10 perform well. There are, however, some limitations regarding the usage of this dataset, which we will briefly reflect upon here, as well as giving directions for future research.

First, the STORM dataset is based on average present-day climate conditions (1980–2017), and as such does not capture climate variability. The climatology represented by STORM may be biased by the phases of multi-decadal variability contained in the 38-year period of record that was used to generate the dataset, which may not be representative for longer timescales. Moreover, the STORM dataset cannot be used to assess climate trends on decadal timescales or the effects of climate oscillations on TCs (e.g. the El Niño Southern Oscillation or the Madden-Julian Oscillation^30^). Future research could study these aspects by e.g. using ensemble runs, or by generating synthetic TCs per oscillation phase.

Second, we used an easily applicable empirical inland decay function^31^ to model the decay of TC wind speeds after landfall, and combine this with a 2D-parametric wind field model^32,33^. This decay function was derived using USA landfalling events, and as such may perform less well elsewhere. Moreover, the function assumes that a TC starts to decay after the TC eye crosses land. In reality, the inland surface winds will decay prior to landfall in response to enhanced surface friction caused by the land mass^34^. The 2D-parametric model does not include the influence of land, and therefore inland wind speeds may be overestimated. The use of a numerical boundary layer model which includes the effects of terrain on the wind field would results in a better representation of the temporal evolution of the TC wind field over land^35^.

Last, the 2D-parametric wind field model used here assumes the asymmetry in the TC wind field arises from background flow. In extratropical regions, however, enhanced wind shear, caused by large-scale background flows or nearby troughs^36^, may also induce asymmetry. Furthermore, STORM does not model the extratropical transition of TCs, so systems in these regions may be represented incorrectly and end-users should therefore pay attention when using this regional data.

### Concluding remarks

We have demonstrated the application of the STORM dataset to generate a novel, open-access dataset of wind speed RPs for all TC basins. We empirically derived RPs at three spatial levels: at basin-level, within 100 km of selected coastal locations, and at 10 km resolution. First, we demonstrated the benefit of using such large synthetic dataset, composed of 10,000 years of TC activity for present-day climate conditions, over using a climatological dataset of 38 years for the calculation of RPs. Compared to the empirically derived RPs, fitting a continuous EV distribution to 38 years of data typically leads to higher max U10, especially for RPs exceeding 100-yr. Second, we calculated RPs for TCs within 100 km of 18 coastal cities, and found that RP-curves differ substantially between locations. Finally, we estimated RPs at 10 km resolution by applying a 2D-wind field model to the synthetic TCs. This dataset is applicable for high-resolution TC wind risk assessments, particularly at the local scale such as Pacific island countries or the Caribbean.

To assess our model performance, we compared the STORM-RPs against those derived from other synthetic models across different spatial scales, and found that results generally agree well. Near basin boundaries, however, RPs can be lower compared to other literature. This is because STORM is run at the basin-scale, and TCs are cut off at the basin boundaries. For regional-scale studies, this issue can be solved by applying STORM using adjusted basin boundaries. On the global scale, we observe similar spatial RP patterns for a Category-1 event compared to literature, but larger spatial differences arise when looking at the distribution of Category-3 RPs in the Southern basins^30^.

In conclusion, this study is unique in that it is the first to estimate (high) RPs at 10 km resolution on a global scale. It represents an important step forward in global TC wind risk assessments, particularly for island countries and TC-scarce regions. Furthermore, this research can contribute to an improved quantification of other TC-induced hazards such as storm surge and precipitation^4–6^. To estimate the RPs of TC-induced storm surges, the 2D-wind fields can be used to force a hydrodynamic model^37^. TC precipitation fields are closely related to max U10^4^ fields and the distance from the eye^35^. These properties can be used to construct a parametric 2D-precipitation field model similar to the parametric wind field model, to assess TC precipitation risk.

## Methods

Our approach is based on estimating the empirical RPs on basis of the synthetic TCs derived from the STORM dataset^12^. This dataset is created using historical data from the International Best Track Archive for Climate Stewardship (IBTrACS^20^) as input dataset for the Synthetic Tropical cyclOne geneRation Model (STORM). The development of this dataset has been described in detail in Bloemendaal, *et al*.^12^. Here, we provide a brief description of the STORM dataset, but for more details on the methodology and validation we direct readers to Bloemendaal, *et al*.^12^.

### Data

The STORM dataset, a global synthetic dataset comprised of 10,000 years of synthetic TCs under present-climate conditions, is used for the calculation of the return periods (RPs). The STORM dataset was generated using STORM. This model takes the following IBTrACS data as input: the latitudinal and longitudinal position of the TC, maximum 10-meter 10-minute average sustained wind speeds (max U10), mean sea-level pressure (MSLP), and the size of the TC eye (Radius to maximum winds; Rmax). Averaged environmental conditions are modeled using monthly-mean sea-surface temperatures (SST) and MSLP fields from ERA5.^38^ From this, autoregressive formulas model consecutive changes in the longitudinal/latitudinal position of the TC (in °), the minimum pressure (in hPa) and the maximum wind speed (in m/s) at every time step during a TC’s lifetime. In addition, STORM also simulates the size of the TC eye, represented via the radius to maximum winds (in km).

STORM is validated in Bloemendaal, *et al*.^12^. Results show that STORM preserves the TC statistics as found in the IBTrACS input dataset, which indicates a good model performance. The average number of both genesis and landfalling events in the STORM dataset, as well as landfall intensity was found to closely correspond (within one standard deviation) to those in the IBTrACS dataset. The largest deviations in max U10 along a TC track were found to be approximately 2 m/s in the STORM dataset compared to the IBTrACS dataset.

### Estimation of return periods and 2D wind field parameterization

Using the 10,000 years of TC activity in the STORM dataset, we empirically calculate the max U10 for different RPs using Weibull’s plotting formula^16^, see Equation 1. The Weibull plotting formula has been demonstrated to be the best performing empirical formula for the estimation of return periods^39^.

1a $${P}_{exc}\left(\overrightarrow{v}\right)=\frac{i}{n+1}\cdot \frac{n}{m}$$

1b $$T\left(\overrightarrow{v}\right)=1/{P}_{exc}(\overrightarrow{v})$$

Here, ${P}_{exc}(\overrightarrow{v})$ represents the exceedance probability ${P}_{exc}$ for a given maximum wind speed $\overrightarrow{v}$ at rank *i*. *n* is the total number of events in the set, and m the total length of the dataset (in years; here, $m=10,000$). The return period $T(\overrightarrow{v})$ is then given as the inverse of ${P}_{exc}$. Because the STORM dataset represents 10,000 years of TC activity, we empirically calculate RPs up to 10,000 years.

To demonstrate the performance of the empirical distribution compared to extreme value distributions (see Results), we fit five continuous extreme value distributions to the STORM dataset. These distributions include the Generalized Extreme Value, Exponential, Gumbel, Weibull, and Pareto distribution. Estimation of the optimal parameters for each of the distributions was done using Python’s *lmoments* package^40^ at 0.1-year intervals, up to 1,000 years. For the estimations of RPs at basin scale and within a 100 km radius, we apply Equation 1 directly to max U10 values in the STORM dataset. However, for assessing RPs of wind speeds at 10 km resolution, we need to convert the TC tracks, that includes the longitudinal/latitudinal position of the TC eye, maximum wind speed, and radius to maximum winds, to 2D-wind fields. For this, we follow the parametric approach of Holland^32^. We follow the same approach as was suggested by Lin and Chavas^33^ and Muis, *et al*.^41^. The Holland B parameter is calculated following Lin and Chavas^33^. The surface wind is converted to a gradient wind using a wind speed reduction factor of 1/0.85^42^. The asymmetry in the surface wind is accounted for by adding the surface background winds to the wind field^33^, which is approximated by the translational speed of the TC. We compute the 2D-wind field using a mesh with 10 km resolution. To optimize computational costs, this calculation is done in parallel using a separate mesh per basin. For each synthetic TC, we store the max U10 at each grid cell whenever max U10 ≥ 20 m/s. Lastly, we apply Equation 1 to the max U10 values at every grid cell to estimate the RPs.

### Basin definitions

The basin definitions used in this paper are adapted from Bloemendaal, *et al*.^12^, see Table 2.

**Table 2:** Basin definitions, as adapted from Bloemendaal, *et al*.^12^.

Basin name	Basin domain
**Eastern Pacific**	5°–60°N 180°-coastline of North America on the North Atlantic
**North Atlantic**	5°–60°N coastline of North America on the Eastern Pacific - 360°
**North Indian**	5°–60°N 30°–100°E
**South Indian**	5–60°S 10°–135°E
**South Pacific**	5–60°S 135°–240°E
**Western Pacific**	5–60°N 100°–180°E

### Saffir-Simpson scale

We use the Saffir-Simpson Scale^26^ as an additional metric to communicate about wind speeds. The categorization on this scale, however, is done using 1-minute average sustained wind speeds, whereas the STORM wind data is given as a 10-minute average value. For this reason, we convert the 1-minute threshold values on the Saffir-Simpson scale to a 10-minute equivalent using a conversion factor of 0.88^21^, see Table 3.

**Table 3:** Tropical cyclone wind speed categorization on the Saffir-Simpson Scale.

Category	Wind speed threshold	
	1-minute average sustained	10-minute average sustained
**Category 1**	64–82 kt	29.0–37.1 m/s
	32.9–42.2 m/s	
**Category 2**	83–95 kt	37.6–43.0 m/s
	42.7–48.9 m/s	
**Category 3**	96–112 kt	43.4–50.7 m/s
	49.3–57.6 m/s	
**Category 4**	113–136 kt	51.1–61.6 m/s
	58.1–70 m/s	
**Category 5**	**≥**137 kt	≥61.6 m/s
	≥70 m/s	

Conversion between 1-minute average sustained wind speeds in knots and in m/s is done using a factor of 0.5144, conversion between 1-minute and 10-minute average sustained wind speeds (in m/s) is done using a conversion factor of 0.88^21^.

**Table 4:** Overview of entries in the “fixed return periods” dataset. Wind speeds are given as 10-meter 10-minute sustained average values.

Parameters		
Name	Unit	Notes
Longitude (lon)	Degrees east	Ranges from 0–360°, with prime meridian at Greenwich
Latitude (lat)	Degrees north	
Return period (rp)	Year	Given at 10, 20, 30 … 100, 200, 300 … 1,000, 2,000, 3,000 … 10,000-yr return period
**Variables**		
**Name (dependency)**	**Unit**	**Notes**
Mean (lat, lon, rp)	m/s	Mean wind speed of 1,000 random realizations, at each return period
Standard deviation (lat, lon, rp)	m/s	Standard deviation in wind speeds of 1,000 random realizations, at each return period
5% confidence interval (lat, lon, rp)	m/s	5% confidence interval value derived from 1,000 random realizations, at each return period
95% confidence interval (lat, lon, rp)	m/s	95% confidence interval value derived from 1,000 random realizations, at each return period

**Table 5:** Overview of entries in the “fixed wind speeds” dataset. Wind speeds are given as 10-meter 10-minute sustained average values.

Parameters		
Name	Unit	Notes
Longitude (lon)	Degrees east	Ranges from 0–360°, with prime meridian at Greenwich.
Latitude (lat)	Degrees north	
Wind speed (u)	m/s	Given at 20, 25, 30.. 75 m/s, and at 29, 37.6, 43, 50.7, and 61.6 m/s to comply with the category thresholds on the Saffir-Simpson scale^26^ (see Methods)
**Variables**		
**Name (dependency)**	**Unit**	**Notes**
Mean (lat, lon, u)	year	Mean return period of 1,000 random realizations, at each wind speed
Standard deviation (lat, lon, u)	year	Standard deviation in return periods of 1,000 random realizations, at each wind speed
5% confidence interval (lat, lon, rp)	year	5% confidence interval value derived from 1,000 random realizations, at each wind speed
95% confidence interval (lat, lon, rp)	year	95% confidence interval value derived from 1,000 random realizations, at each wind speed

**Table 6:** Overview of entries in the “cities” dataset.Wind speeds are given as 10-meter 10-minute sustained average values.

Entry	Column name	Notes
1	City	Name of city
2	Country	Name of country
3	Latitude	Latitudinal coordinates of capital city as given in Google Maps (maps.google.com).
4	Longitude	Longitudinal coordinates of capital city as given in Google Maps (maps.google.com), ranging from 0–360° with prime meridian at Greenwich.
5–31	Return period	On “Fixed return periods”-tab: maximum wind speed (m/s) at given return period
5–20	Wind speed	On “Fixed wind speeds”-tab: return period (yr) at given wind speed. Wind speeds are given at 5 m/s intervals and at values corresponding to Category thresholds on the Saffir-Simpson Scale, see Methods.

**Table 7:** Overview of entries in the “islands” dataset. Wind speeds are given as 10-meter 10-minute sustained average values.

Entry	Column name	Notes
1	City	Name of capital city
2	Country	Name of Island Country
3	Latitude	Latitudinal coordinates of capital city as given in Google Maps (maps.google.com).
4	Longitude	Longitudinal coordinates of capital city as given in Google Maps (maps.google.com), ranging from 0–360° with prime meridian at Greenwich.
5–31	Return period	On “Fixed return periods”-tab: maximum wind speed (m/s) at given return period
5–20	Wind speed	On “Fixed wind speeds”-tab: return period (yr) at given wind speed. Wind speeds are given at 5 m/s intervals and at values corresponding to Category thresholds on the Saffir-Simpson Scale, see Methods.

## Data Availability

The STORM dataset was developed in Bloemendaal, *et al*.^12^ and is publicly accessible via the 4TU.Centre for Research Data repository^43^. This paper is accompanied by four different datasets: (i) The “fixed return periods” dataset, with wind speed estimates (m/s) for a predefined range of return periods for every longitude/latitude position of the 10 km grid per basin (demonstrated in Fig. 3, see Table 4); (ii) The “fixed wind speeds” dataset, with return period estimates (yr) for a predefined range of wind speeds for every longitude/latitude position of the 10 km grid per basin (demonstrated in Fig. 4, see Table 5); (iii) The “cities” dataset, with return periods (yr) for a predefined range of wind speeds (m/s) and wind speeds for a predefined range of return periods, occurring with 100 km of the respective city (demonstrated in Fig. 2; see Table 6). (iv) The “island” dataset, with return periods (yr) for a predefined range of wind speeds (m/s) and wind speeds for a predefined range of return periods, occurring with 100 km of the capital city of the respective island. We included the Small Island Developing States and a set of other islands in this dataset (63 islands in total; see Table 7). This dataset has not been demonstrated in the paper, but application is similar to the “cities” dataset. Datasets (i) and (ii) are netCDF4 (.nc)-files, dataset (iii) and (iv) are excel-files (.xlsx). Each of these datasets can be found at the 4TU.Centre for Research Data repository^44^–^47^.

## Online-only Table

**Online-only Table 1:** Comparison of maximum 10-minute 10-meter average sustained wind speeds (max U10) at different locations and for different return periods (RP) between STORM and models used in other literature. Wind speeds are given in m/s, return periods in years.

Location	Spatial scale	Max U10 reference (m/s)	Max U10 STORM (m/s)	RP reference (yr)	RP STORM (yr)	Absolute difference in max U10 (m/s)	Relative difference in max U10 (%)	Evaluation of RP	Research setup, Reference
Mumbai, India	150 km radius^1^	Category 1		49–97	66	—		Within range	CHAZ model^29^ and MIT model^9^, Sobel, *et al*.^24^
		Category 3		~500	138	—		Lower than literature	
	10 km grid cell	Category 1		224–236	95	—			
		Category 3		~3000 –>10.000	550	—			
Honaira, Solomon Islands	10 km grid cell	27.2^2^	31.4	100		4.2	15.4	—	AIR South Pacific Risk Model, Commonwealth of Australia^28^
Suva, Fiji		41.2^2^	40.8			0.4	−1.0		
Palikir, Federal States of Micronesia		36.5^2^	23.4			13.1	−35.9		
Port Vila, Vanuatu		46.8^2^	40.5			6.3	−12.8		
Port Moresby, Papua New Guinea		26.7^2^	23.7			3.0	−11.2		
Dili, Timor-Leste		16.6^2^	18.4			1.8	10.8		
Koror, Palau		37.9^2^	28.4			9.5	−25.1		
Tongatapu, Tonga		42.2^2^	38.7			3.5	−8.3		
Alofi, Niue		40.3^2^	39.8			0.5	−1.2		
Atiu, Cook Islands		43.1^2^	35.5			7.8	−17.6		
Apia, Samoa		40.5^2^	43.2			2.7	6.7		
Vaiaku, Tuvalu		27.3^2^	28.4			1.1	4.0		
Charleston (NC), USA	100 km radius	51.8	53.8	100		2	3.9	—	Hurricane Risk Calculator^45^, Ellis, *et al*.^46^
The Battery (NY), USA	10 km grid cell	27.3^2^	30	100		2.7	9.9	—	MIT model^9^, Garner, *et al*.^47^
		36.1^2^	40	1,000		3.9	10.8	—	
Darwin, Australia	100 km radius	65^3^	47	500		18	−38.3	—	MIT model^9^, Cook and Nicholls^27^
Townsville, Australia		52^3^	49			3	−5.8		
Port Hedland, Australia		54^3^	54			0	0		

^1^In this specific case, the STORM return period analysis has been re-run at 150 km radius instead of the regular 100 km used in the other cases.

^2^Converted from 1-min sustained wind speeds to 10-min average maximum wind speeds using a conversion factor of 0.88^9^.

^3^Converted from 3-sec wind gusts to 10-min average maximum wind speeds using a conversion factor of 0.66^9^.

## Code availability

All Python algorithms used to generate the datasets described here are publicly available via https://github.com/NBloemendaal/STORM-return-periods. The STORM Python script can be found on https://github.com/NBloemendaal/STORM and the data-preprocessing is available via https://github.com/NBloemendaal/STORM-preprocessing.
